# A GPU-Based Implementation of the Firefly Algorithm for Variable Selection in Multivariate Calibration Problems

**DOI:** 10.1371/journal.pone.0114145

**Published:** 2014-12-10

**Authors:** Lauro C. M. de Paula, Anderson S. Soares, Telma W. de Lima, Alexandre C. B. Delbem, Clarimar J. Coelho, Arlindo R. G. Filho

**Affiliations:** 1 Institute of Informatics, Federal University of Goiás - UFG, Goiânia, GO, Brazil; 2 Institute of Mathematical Sciences and Computing, University of São Paulo - USP, São Carlos, SP, Brazil; 3 Computer Science Department, Pontifical Catholic University of Goiás - PUC Goiás, Goiânia, GO, Brazil; 4 Department of System and Control, Technological Institute of Aeronautics - ITA, São José dos Campos, SP, Brazil; National Institute of Environmental and Health Sciences, United States of America

## Abstract

Several variable selection algorithms in multivariate calibration can be accelerated using Graphics Processing Units (GPU). Among these algorithms, the Firefly Algorithm (FA) is a recent proposed metaheuristic that may be used for variable selection. This paper presents a GPU-based FA (FA-MLR) with multiobjective formulation for variable selection in multivariate calibration problems and compares it with some traditional sequential algorithms in the literature. The advantage of the proposed implementation is demonstrated in an example involving a relatively large number of variables. The results showed that the FA-MLR, in comparison with the traditional algorithms is a more suitable choice and a relevant contribution for the variable selection problem. Additionally, the results also demonstrated that the FA-MLR performed in a GPU can be five times faster than its sequential implementation.

## Introduction

Multivariate calibration refers to construction procedure of a mathematical model that establishes the relationship between the properties measured by a instrument and the concentration of a sample to be determined [1]. The building of a model from a subset of explanatory variables usually involves two conflicting objectives:

- Extracting as much information from a measured data with many possible independent variables;
- Decreasing the cost of obtaining data by using the smallest set of independent variables that results in a model with high accuracy and low variance.

The balance between these two commitments is achieve using variable selection techniques. The application of multivariate calibration had a breakthrough and nowadays are very popular [2]. One of the most interesting features of modern instrumental methods is the number of variables that can be measured in a single sample. Recently, devices as spectrophotometers have generated large amount of data with thousands of variables. As a consequence, the development of efficient algorithms for variable selection is important in order to deal with data even larger [3]–[5]. Furthermore, a high-performance computing framework can significantly contribute to efficiently construct an accurate model [6]. In this context, this paper presents an implementation of a modified Firefly Algorithm (FA) for variable selection in multivariate calibration problems [4], [7], [8]. The FA is a metaheuristic inspired by the flashing behaviour of fireflies [9].

Several works have used FA to solve many types of problems. For instance, Yang [10] provided a detailed description of a new FA for multimodal optimization applications. Lukazik and Zak [11] provided an implementation of the FA for constrained continuous optimization. Yang [12] showed the use of the FA for nonlinear design problems. Senthilnath *et al.* [13] used the FA for clustering on benchmark problems and compared its performance with other nature-inspired techniques. Gandomi *et al.* [14] used the FA for mixed-continuous and discrete-structural optimization problems. Jati *et al.* [15] applied the FA for travelling salesman problem. Banati and Monika [16] proposed a new feature selection approach that combines the Rough Set Theory with the nature-inspired FA to reduce the dimensionality of data containing large number of features. Horng [17] proposed a new method based on the FA to construct the codebook of vector quantization for image compression. Finally, Abdullah *et al.* [18] introduced a new hybrid optimization method incorporating the FA and the evolutionary operation of the differential evolution method. In all these works, the experimental results showed that the FA scores over other algorithms in terms of computing time and optimality.

This paper also uses a Graphics Processing Unit (GPU) to parallelize the computation of the vector of regression coefficients in the problem of Multiple Linear Regression (MLR). GPUs can be employed to improve performance of computing applications usually handled by a Central Processing Unit (CPU) [5]. Husselman and Hawick [19], [20] are the only works we have found so far that present a GPU-based implementation of a FA. Moreover, estimates from the proposed FA (FA-MLR) are compared with predictions from the following traditional algorithms: Successive Projections Algorithm for MLR (SPA-MLR) [7], Genetic Algorithm for MLR (GA-MLR) [21], [22], Partial Least Squares (PLS) [23] and Bayesian Variable Selection (BVS) [24]. In addition, it is used three others criterions to determine the predictive ability of MLR models. The generalization ability of the models is also evaluated by adding artificial measurement noise to the independent variables.

The remaining of the paper is organized as follows. Section “Background” describes the multivariate calibration problem, the multicollinearity and variable selection problems, and the original FA. The FA-MLR and the processing on a GPU are presented in Section “The FA for Variable Selection”. Section “Materials and Methods” describes the material and methods used in the experiments. Results are discussed in Section “Results and Discussion”. Finally, Section “Conclusion” shows the conclusions of the paper.

## Background

### Multivariate Calibration

The multivariate calibration model provides the value of a quantity 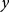 based on values measured from a set of explanatory variables 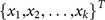 [25], [26]. The model can be defined as:
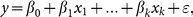
(1)where 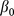, 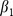,…, 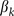, 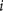 = 1, 2,…, 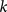, are the coefficients to be determined, and 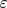 is a portion of random error.

A simple model to obtain the coefficients in Equation (1) based on calculation of partial least squares is known as MLR, which is a statistical technique used to build models describing reasonably relationships between several explanatory variables of a given process [27], [28]. This technique requires the number of observations greater than the number of variables. Nevertheless, the opposite may occur for some applications (more variables than samples) [8]. For instance, in problems involving spectrometric determination of a physical or chemical quantity the explanatory variables correspond to measurements taken at various wavelengths [29].

Equation (2) shows how the regression coefficients may be calculated using the Moore-Penrose pseudoinverse [30]:
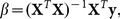
(2)where 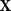 is the matrix of samples and independent variables (collected by instruments used for construction of multivariate calibration models), **y** is the vector of dependent variables (or property of interest obtained in laboratory, which serves as a parameter for model calibration), and 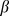 is the vector of regression coefficients.

As shown in Equation (3), predictive ability of MLR models comparing predictions with reference values for a test set from the squared deviations is calculated by Root Mean Squared Error of Prediction (RMSEP):
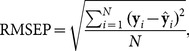
(3)where **y** is the reference value of the property of interest, 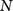 is the number of observations, and 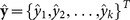 is the estimated value calculated as:



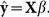
(4)Another criteria to determine the predictive ability of MLR models that has been used is the Mean Absolute Percentage Error (MAPE) [31]. MAPE is a relative measure which express errors as a percentage of the actual data and is defined as:
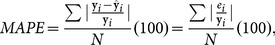
(5)where 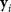 is the actual data at variable 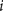, 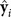 is the forecast (using some model/method) at variable 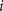, 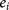 is the forecast error at variable 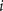, and 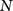 is the number of observations (or samples) used in computing the MAPE.

The biggest advantage of MAPE is that it provides an easy and intuitive way of judging the extent (or the importance) of errors. Furthermore, percentage errors are part of the every day language making them easily and intuitively interpretable [31]. This measure is widely used in forecasting as a basis of comparison. It can be used to measure how high or low are the differences between the predictions and actual data in regression models in a similar way to forecasting problems.

In statistics, there is also a technique called Predicted Residual Sums of Squares (PRESS) proposed by Allen [32]. PRESS is a useful statistic for comparing different models and is based on the leave-one-out technique [33]. It is also known as a form of cross-validation used in regression analysis to provide a summary measure of the fit of a model to a sample of observations that were not themselves used to estimate the model [32]. The PRESS also may be used as a measure of predictivity to compare and select the best model. However, one of the main problems of cross-validation techniques is their computational cost, which may become extremely higher and unviable [34]. Equation (6) shows how the PRESS is calculated:
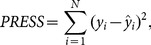
(6)where 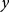 is a real value of concentration obtained by laboratorial methods, 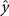 is the result of Equation (4) applied to measures of new observations (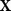 measures), and 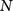 is the number of observations.

### Multicollinearity Problem and Variable Selection

In prediction problems with regression model having many variables, most of them may not contribute to improve prediction precision. The selection of a reduced set with variables that positively influence in the regression model is important in order to improve the efficiency of algorithms for construction MLR models. Moreover, the identification of a small set of variables that are explanatory is usually desired in regression problems [3]. The problem of determining an appropriate equation associated to a subset of independent variables depends on the criteria used to: 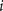) analyze the variables, 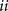) select the subset and 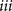) estimate the coefficients in Equation (2).

There are some model (or variable) selection criterias in the literature [35]. An approach is the use of information criteria such as Akaike Information Criteria (AIC) proposed by Akayke [36] or the Bayesian Information Criteria (BIC) proposed by Schwarz [37]. Equation (7) and (8) show how AIC and BIC may be calculated, respectively:
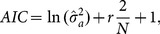
(7)




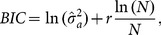
(8)where 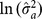 denotes the maximum likelihood estimate of 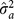, 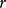 denotes the number of parameters estimated in the model, including a constant term, and 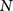 is the number of samples [35].

In the information criteria approach, models that yield a minimum value for the criterion are to be preferred. Generally, the AIC and BIC values are compared among various models as the basis for selection of the model. However, a disadvantage of this approach is that several models may have to be estimated by maximum likelihood, which is expensive and may require a huge computational effort [35].

In this context, a Firefly Algorithm is used in this paper to solve the problem of variable selection for the multivariate calibration problems, as described in Section “The Proposed FA for Variable Selection”.

### Firefly Algorithm

Nature-inspired metaheuristics have shown to be powerful in solving various types of problems. The FA is a recently developed optimization algorithm proposed by Yang [9], [10]. This algorithm is based on the idealized behaviour of the flashing characteristics of fireflies. The FA simulates the attraction system of real fireflies. They produce luminescent flashes as a signal system to communicate with other fireflies, especially to prey attraction [16]. Algorithm 1 (Table 1) shows a pseudocode for the original FA.

**Table 1:** Algorithm 1. Original Firefly Algorithm.

1. Initialize a population of fireflies 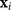 , 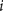 = 1, 2,…, 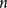
2. Calculate objective function f( 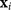 ) for each firefly
3. Define light absorption coefficient 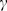
4. **while** 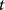 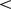 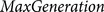
5. **for** 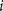 = 1: 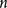
6. **for** 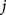 = 1: 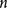
7. Light intensity 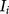 at 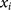 is determined from f( 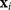 )
8. **if** ( 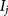 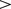 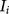 )
9. Calculate the attractiveness between 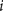 and 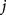 which varies with distance 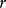 via 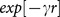
10. Move firefly 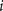 towards 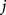 in all 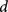 dimensions according to the attractiveness between 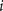 and 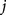
11. **end if**
12. Evaluate the new fireflies and update light intensities
13. **end for** 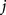
14. **end for** 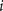
15. Rank the fireflies and find the current best
16. **end while**
17. Postprocess results

In the Algorithm 1 (Table 1), there are two important issues: the variation of light intensity and the attractiveness formulation. For simplicity, one can assume that the attractiveness of a firefly is determined by its brightness or light intensity which is associated with the encoded objective function [10]. The brightness 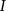 of a firefly at a particular location **x** can be chosen as 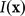 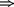 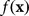. However, the attractiveness 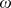 is relative, which should be seen in the eyes of the beholder or judged by the other fireflies. Then, it should vary with the distance 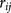 between firefly 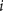 and firefly 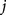. As light intensity decreases with the distance from its source, and light is also absorbed in the media, so the attractiveness should vary with the degree of absorption [12]. In the simplest form, the light intensity 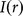 varies with the distance 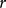 monotonically and exponentially as shown by Equation (9):
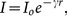
(9)where 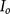 is the original light intensity and 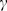 is the light absorption coefficient.

As a firefly's attractiveness is proportional to the light intensity seen by adjacent fireflies, one can define the attractiveness 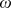 of a firefly by:
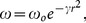
(10)where 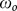 is the attractiveness at 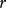 = 0.

It is worth pointing out that the exponent 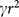 can be replaced by other functions such as 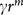 when 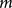 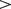 0 [12].

The distance between any two fireflies is calculated using Cartesian distance in Equation 11.
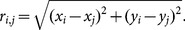
(11)


The firefly 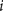 is attracted to brighter firefly 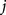 and its movement is determined by
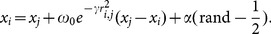
(12)


In Equation (12), 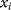 is the current position or solution of a firefly and the 
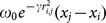
 is attractiveness of a firefly to seen by adjacent fireflies. The 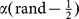 is a firefly's random movement. The coefficient 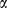 is a randomisation parameter determined by the problem of interest with 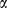 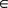 [0,1], while rand function is a random number obtained from the uniform distribution. Without the random movement, the reies would possibly be attracted to a rey that is not necessarily the brightest. The solution would be restricted to a local minima, directly toward the best solution in the local search space. Using the randomization term, the search over small deviations makes it possible to escape from local minima, having a higher chance of nding the global minimum of the function.

For most cases related in others works, they take 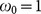, 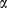 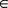 [0,1] and 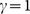. The parameter 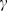 characterizes the variation of attractiveness, and its value is crucially important in determining the speed of the convergence and how the FA algorithm behaves. In most applications, it typically varies from 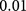 to 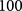.

According with Yang [38], the algorithm is swarm-intelligence-based, so it has the similar advantages that other swarm-intelligence-based algorithms have. In fact, a simple analysis of parameters suggest that some particle swarm optimization (PSO) variants such as Accelerated PSO are a special case of firefly algorithm when 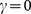. However, according with Yang [38], firefly has two major advantages over other algorithms: automatical subdivision and the ability of dealing with multimodality. First, FA is based on attraction and atractiveness decrease with distance. This leads to the fact that the whole population can automatically subdivide into subgroups, and each group can swarm around each mode or local optimum. Second, this subdivision allows the fireflies to be able to find all optima simultaneously if the population size is sufficiently higher than the number of modes.

### The Proposed Multiobjective FA for Variable Selection

Based on the success of the works cited in “Introdution”, one found out that FA can be used for selection of variables to solve multivariate calibration problems. Thus, this paper presents a FA for variable selection using MLR (FA-MLR). Algorithm 2 (Table 2) shows a pseudocode for the FA-MLR.

**Table 2:** Algorithm 2. Proposed FA-MLR.

1. **Parameters**: 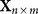 , 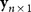 , where 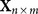 is a matrix of samples by their corresponding variable values, 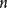 is the number of rows, 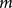 is the number of columns and 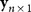 is a vector of dependent variables
2. 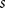 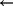 the number of fireflies, determined by experimental analysis.
3. **for** 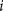 = 1: 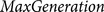
4. Generate randomly a population of 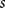 fireflies, where each firefly is a set of 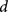 indices for 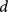 columns of 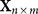 , 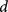 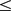 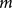
5. Compute Equation (2) for each firefly (when using Algorithm 3 it is performed on the GPU)
6. Compute the number of variables used in the model
7. Calculate Equation (3) determining the light intensities of each firefly
8. If 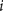 -th firefly dominates 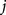 -th firefly
9. Move firefly 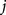 towards 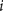 using Equation (12)
10. Rank the fireflies and find the current best
11. **end for** 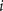
12. Postprocess results, that is, calculate the prediction error for the best firefly and visualize the selected variables indicated by it.

The original formulation of FA uses the evaluation of a single objective. However, previous works [8], [39], [40] in multivariate calibration showed that while monoobjective formulation use a bigger number of variables, multiobjective algorithms use fewer variables with the less prediction error. Thus, in this work, we proposed a multiobjective optimization implementation. One classical way is to combine all objectives into a single objective so that algorithms for single objective optimization. Another way is to extend the firefly algorithm to produce Pareto optimal front directly. By extending the basic formulation of FA, we developed the following multiobjective firefly algorithm, based on [41], summarized in Algorithm 2 (Table 2).

In the multiobjective formulation of FA, the choice of current best is done in two steps. First, only the non-dominated solutions are selected. In mathematical terms, a feasible solution 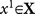 is said dominate another solution 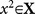 if:

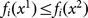
 for all indices 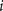 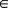 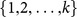 and

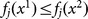
 for at least one index 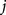 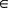 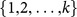

A solution 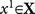 is called Pareto optimal, if there does not exist another solution that dominates it. Among non-dominates solutions, we applied the multiobjective decision maker method described in [8] to choice the current best (step 10).

### Numerical Example

For illustration, let's consider a short variable selection problem with just five variables available and just three fireflies. Will be used, the first five variables available and the first three samples from matrix 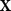 and the first three samples from vector 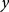 described in Materials and Methods Section, using 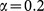, 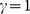 and 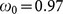. First, the fireflies are uniformly distributed random numbers in the range 0 to 1.

Firefly 1  =  0.95 0.48 0.45 0.44 0.92,

Firefly 2  =  0.23 0.89 0.01 0.61 0.73,

Firefly 3  =  0.60 0.76 0.82 0.79 0.17.

The variable selection is a binary problem, so each firefly must be encoded. Each variable information major than 0.5 is encoded to 1 (variable will be used in the regression model) and less or equal 0.5 is encoded 0 (variable will not be used in the regression model).

Encoded Firefly 1  =  1 0 0 0 1,

Encoded Firefly 2  =  0 1 0 1 1,

Encoded Firefly 3  =  1 1 1 1 0.

Now, each firefly will be evaluated using the Equation 2. This equation indicates the error of prediction. In Equation 2, just the columns of 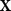 indicated by firefly will be used in the regression. In our example the brightness is 6.82 10.97 9.22 for each firefly. Now, we compared all firefly each other, for example, the firefly 2 has major light intensity than firefly 1, so we must move firefly 1 toward firefly 2. For this proposes, first we must calculate the distance between the fireflies using Equation 11.


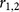
  =  0.71 0.40 0.43 0.17 0.18

Using the distance between the fireflies, we can calculate the attractiveness using Equation 10. As a result, we updated firefly 1 and the encoded firefly 1. Worth noting that, the updated firefly 1 excluded the first variable used in the original solution and now use the second and fourth variables available after ‘to walk’ toward firefly 2.

Updated Firefly 1  =  0.44 0.75 0.02 0.53 0.67→

Updated Encoded Firefly 1  =  0 1 0 1 1

The iteration is repeated until all solutions have been updated. The updates allow the solutions to move towards to the current optimal solution. The solution that produces the best fitness (in this case the minor RMSEP value) is selected as the global best solution.

### Using a GPU with the CUDA-MATLAB integration for the FA-MLR

GPUs are microprocessors developed with a flow-oriented technology, optimized for calculations of data-intensive, where many identical operations can be performed in parallel on different data [42]. Graphics devices currently available represent a high performance computer hardware, flexible by enabling the execution of non-graphical applications [5]. While the current multicore architectures have two, four or eight cores, GPUs have hundreds or even thousands of processing cores [5]. GPUs can implement many parallel algorithms directly using graphics hardware and the current trend is to include in each new generation a significant number of additional cores [43].

Correspondingly the evolution of hardware, new programming models have been developed. Among them stand out Compute Unified Device Architecture (CUDA) [42] and Open Computing Language (OpenCL) [44]. In both, due to the availability of Application Programming Interface (API) to the programmer the implementation of efficient parallel applications is facilitated [5]. CUDA was the first architecture and programming interface, created by 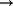 in 2006 to allow GPUs could be used for a wide variety of applications [42].

Recently, using MATLAB for GPU computing can accelerate the applications more easily than by using the CUDA-C programming language [45]. This is possible because of the existence of MATLAB plug-in for CUDA (Parallel Computing Toolbox - PCT). Thus, with the familiar MATLAB language one can take advantage of the CUDA GPU computing technology [46].

Developments for GPUs using the PCT is in general easier and faster than using CUDA-C language, since several aspects of parallelization design are performed by the PCT [47], [48]. Furthermore, it is important to note that the PCT requires an 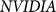 graphics card.

Step 5 (that calculates the regression coefficients) in Algorithm 2 (Table 2) can be executed on a GPU. The parallel execution can be accomplished through MATLAB built-in functions. Algorithm 3 (Table 3) shows the pseudocode for Step 5 using a 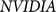 GPU.

**Table 3:** Algorithm 3. Step 5 of the FA-MLR.

1. **Parameters**: 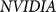 , 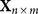 and fireflies 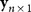 ,…, 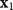
2. **for** 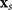 = 1: 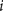
3. Obtain submatrix 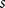 that contains only 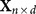 columns of 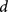 indexed by 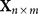
4. Allocate matrices 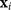 and 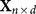 and vector 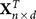 on the GPU memory
5. Calculate Equation (2) on the GPU
6. **end for** 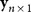

## Materials and Methods

The Firefly algorithm was implemented using 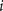, 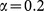 and 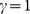. We have used for RMSEP, MAPE, PRESS, AIC and BIC comparison the Successive Projections Algorithm for MLR (SPA-MLR) [7], the standard Genetic Algorithm for MLR (GA-MLR) [21], the Partial Least Squares (PLS) [23] and the Bayesian Variable Selection (BVS). The BVS code was obtained at the following URL: https://github.com/pcarbo/varbvs [24].

Proposed by Araújo *et al.* [49], the goal of SPA-MLR is to select a subset of variables with low collinearity that allow the construction of a MLR model with a capacity of adequate prediction. SPA-MLR comprises three main phases. Phase 1 consists of projection operations carried out on the matrix of instrumental responses (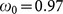). These projections are used to generate chains of variables with successively more elements. Each element in a chain is selected in order to display the least collinearity with the previous ones. In Phase 2, candidate subsets of variables are extracted from the chains and evaluated according to the predictive performance (RMSEP) of the resulting MLR model. Such a performance can be assessed by using cross-validation or a separate validation set. Finally, Phase 3 consists of a variable elimination procedure aimed at improving the parsimony of the model.

In GA-MLR, the RMSEP guide the evaluation of a subset of variables used in the calibration model and allows us to choose models more suitable to prediction [21]. Genetic algorithms (GA) are a global search heuristic inspired on the natural evolution of species and in the natural biological process. Basically, a GA creates a population of possible solutions to the problem being solved and then submit these solutions to the evolution process. Genetic operators are applied to transform the population in every generation, in order to created better individuals. The main operators responsible for the population diversification well known in the literature are crossover (or recombination) and mutation.

Partial Least Squares (PLS) is a technique that generalizes and combines features from principal component analysis and MLR [30]. It is a statistical method that bears some relation to principal components regression. Instead of finding hyperplanes of minimum variance between the response and independent variables, it finds a linear regression model by projecting the predicted variables and the observable variables to a new space. Because both the 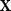 and 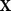 data are projected to new spaces, the PLS family of methods are known as bilinear factor models.

Bayesian Variable Selection (BVS) is a tool to variable selection in MLR used for tackling many scientific problems [24]. In the BVS, the model selection problem is transformed to the form of parameter estimation: rather than searching for the single optimal model, a Bayesian will attempt to estimate the posterior probability of all models within the considered class of models. A probability distribution is first assigned to the dependent variable through the specification of a family of prior distributions for the unknown parameters in the regression model. For each regression coefficient subject to deletion from the model, the prior distribution is a mixture of a point mass at 0 and diffuse uniform distribution elsewhere.

The Kennard and Stone [50] algorithm was applied to the resulting spectra to divide the samples into calibration, validation and prediction sets with 389, 193 and 193 samples, respectively. The validation set was employed to guide the selection of variables in SPA-MLR and GA-MLR. The prediction set was only employed in the final performance assessment of the resulting MLR models. In the PLS study, the calibration and validation sets were joined into a single modeling set, which was used in the leave-one-out cross-validation procedure. The number of latent variables was selected on the basis of the cross-validation error by using the F-test criterion of Haaland and Thomas [23]. The prediction set was only employed in the final evaluation of the PLS model.

All calculations were carried out by using a desktop computer with an Intel Core i7 2600 (3.40 GHz), 8 GB of RAM memory and a 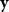 GeForce GTX 550Ti graphics card with 192 CUDA cores and 2 GB of memory config. The Matlab 8.1.0.604 (R2013a) software platform was employed throughout.

We present two studies, one simulated and a real problem, to present the proposed algorithm. The details of the data sets are presented below.

### Simulated Data Set

The simulated data set used in this work was generated using the matlab code showed by *S1 Source Code*. The algorithm uses a random numbers generation for building the matrix 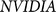 (independents variables). The seed of the random generator was setted with value 0 (zero), thus, the experiment can be reproduced in any computer. To generate the matrix responses 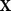, we choose a number of variables from 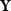 to be correlated with 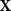 (dependents variables). The values of 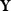 are generated using random weights applied over the variables chosen. We create two scenarios: the first five variables are randomly chosen to be correlated with 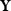 and in the second, ten variables are used. The challenge of variable selection algorithm is to find the variables used to generate 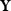. These variables explain the 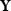 variability and the best result can be obtained selecting only these variables.

### Real Data Set

The real dataset employed in this work consists of whole grain wheat samples, obtained from vegetal material from occidental Canadian producers. The standard data were determined at the Grain Research Laboratory as in work of [7], [5] and [51]. The data set for the multivariate calibration study consists of 1090 Near-Infrared (NIR) spectra of whole-kernel wheat samples, which were used as shoot-out data in the 2008 International Diffuse Reflectance Conference (http://www.idrc-chambersburg.org/shootout.html).

Protein content was chosen as the property of interest (matrix 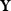). The spectra were acquired in the range 400–2500 nm with a resolution of 2 nm (matrix 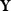). In this work the NIR region in the range 1100–2500 nm was employed. In order to remove undesirable baseline features, first derivative spectra were calculated by using a Savitzky-Golay filter with a 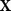 order polynomial and an 11-points window [52].

The reference values of protein concentration in samples of wheat were determined in the laboratory by the Kjeldahl method [53]. This method uses the destruction of organic matter with concentrated sulfuric acid in the presence of a catalyst and by the action of heat, with subsequent distillation and titration of nitrogen from the sample. The use of indirect instrumental methods such as NIR spectroscopy and mathematical models such as MLR allow the protein to be determined without destruction of the sample.

## Results and Discussion

### Simulated Study

In the simulated study, the challenge of the variable selection algorithms is to find the variables in 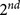 that explain the 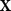 variance. We did two studies, one with five variables generating 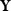, and other with ten variables generating 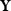.

Table 4 presents the results of all algorithms in the simulated data. As can be seen, the FA-MLR has the minor value of RMSEP, MAPE and PRESS, in both studies, using five and ten variables generating 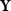. This measures indicates better prediction ability using the variables selected by FA. FA-MLR use less variables than SPA-MLR and BVS. According with AIC and BIC measure, FA-MLR has better parsimony between predictive capacity and number of variables in the model. In some applications the predictive ability is critical, in others the number of variables used or even the parsimony between both.

**Table 4:** Results of the FA-MLR, SPA-MLR, GA-MLR, PLS and BVS, in simulated data.

	Number of Variables	RMSEP	MAPE	AIC	BIC	PRESS
Five variables generating 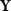						
PLS	4	1.11	3.7%	1279	1330	29.29
SPA-MLR	2	1.05	3.53%	1005	1020	26.82
GA-MLR	26	1.85	4.0%	1981	2013	45.42
BVS	4	0.98	3.4%	1201	1301	21.02
FA-MLR	3	0.97	3.3%	956	972	19.98
Ten variables generating 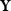						
PLS	4	1.91	3.5%	1054	1093	69.05
SPA-MLR	4	1.75	3.2%	1004	1021	61.26
GA-MLR	35	2.01	4.80%	1791	1807	74.36
BVS	7	1.95	3.3%	1149	1201	70.95
FA-MLR	3	1.67	3.1%	901	933	57.5

Table 5 shows the variable selected by each one of the algorithms. For the first case, the variables used to generating 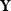 is 22, 32, 34, 40 and 99. For the second case, the variables used were 4, 11, 39, 46, 48, 66, 67, 69, 85 and 189. According with results, for the two cases SPA-MLR and FA-MLR are the more conservative algorithm. SPA-MLR selected just two variables (32 and 99) for the first case, but did not select the other three variables correlated with the response matrix 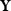. FA-MLR selected three variables (22, 32 and 40), all correlated whit the response matrix 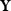, generating a prediction error lower than SPA-MLR. In the second case, SPA-MLR selected three variables (11, 39 and 85) correlated with the response and one wrong variable. FA-MLR selected three variables (4, 46 and 189) all of them related with the response variable. Although the SPA-MLR algorithm use a elimination procedure, described in [54], the multiobjective formulation of FA-MLR was able to balance between prediction error and number of variables. The results of AIC and BIC show that FA-MLR algorithm has better parsimony of MLR models than models builded by SPA-MLR.

**Table 5:** Variable Selected by the Algorithms.

Algorithm	Variables Found
**Five variables generating** 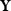	
PLS	-
SPA-MLR	**32**, **99**
GA-MLR	**22**, 23, 29, 35, 38, 39, 46, 47, 55, 66, 69, 71, 78, 84, 90,
	111, 127, 129, 136, 140, 158, 167, 174, 176, 188, 193
BVS	**22**, 31, **99**, 144
FA-MLR	**22**, **32**, **40**
**Ten variables generating** 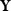	
PLS	-
SPA-MLR	**11**, **39**, **85**, 99
GA-MLR	2, 7, 17, 17, 23, 26, 29, 32, 35, 38, **39**, 40, 42, **46**, 47, 52, 58, 66, 69,
	71, 78, 84, **99**, 111, 127, 129, 136, 140, 158, 167, 174, 176, 181, 188, 193
BVS	**4**, **11**, 31, 99, 158, 167, **189**
FA-MLR	**4**, **46**, **189**

The variables selected that were used to generating 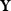 are marked with bold font.

The best result of RMPSEP, MAPE and PRESS were obtained by FA-MLR in both cases. The algorithm selected more variables correlated with the response matrix (3 in both cases) reducing the prediction error. FA-MLR works individually each firefly and finds a better position for itself in consideration with its current position as well as the position of other fireflies. Thus, it escapes from the local optima and finds a global optimum is less number of iterations. However, the algorithm requires a correct adjustment of the parameters, while SPA-MLR, PLS and BVS not.

The worst result was obtained by GA-MLR algorithm. It selected much wrong variables and few variables correlated with the response matrix. The model produce by this algorithm is large and with low prediction ability. PLS uses linear transformation of original variables to build new latent variables. In this process is not possible separate the original variables used, thus, we do not have the results of which original variables used to build the regression model.

### Real Problem

**Figure 1:**
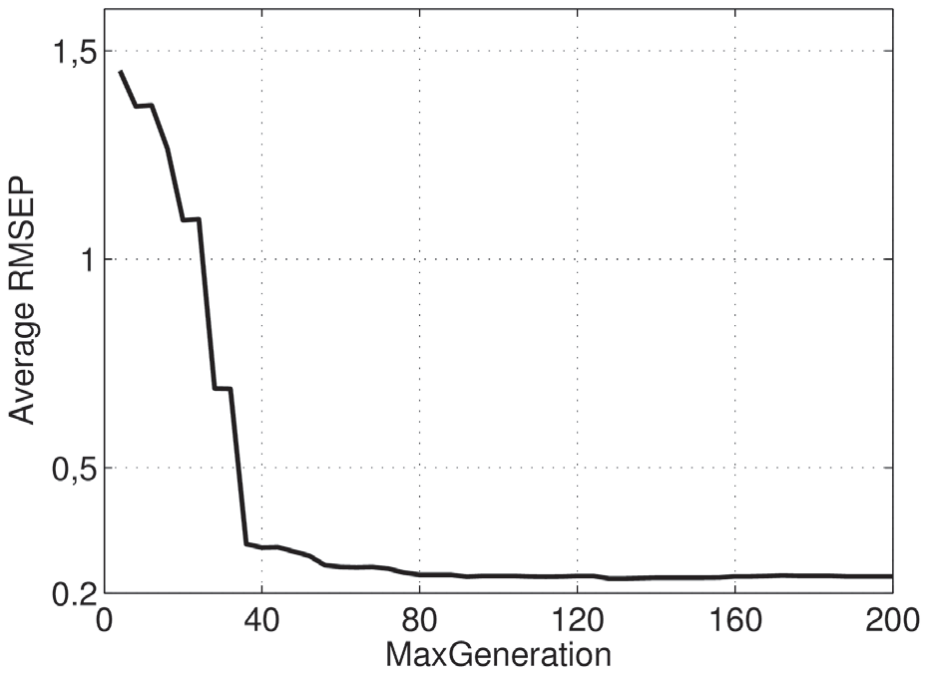
Behavior of average RMSEP versus 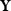.

Fig. 1 shows that the FA-MLR is able to perform the reduction of the RMSEP as the iterations are performed. The curve in the graph refers to the average error of all the fireflies.

**Figure 2:**
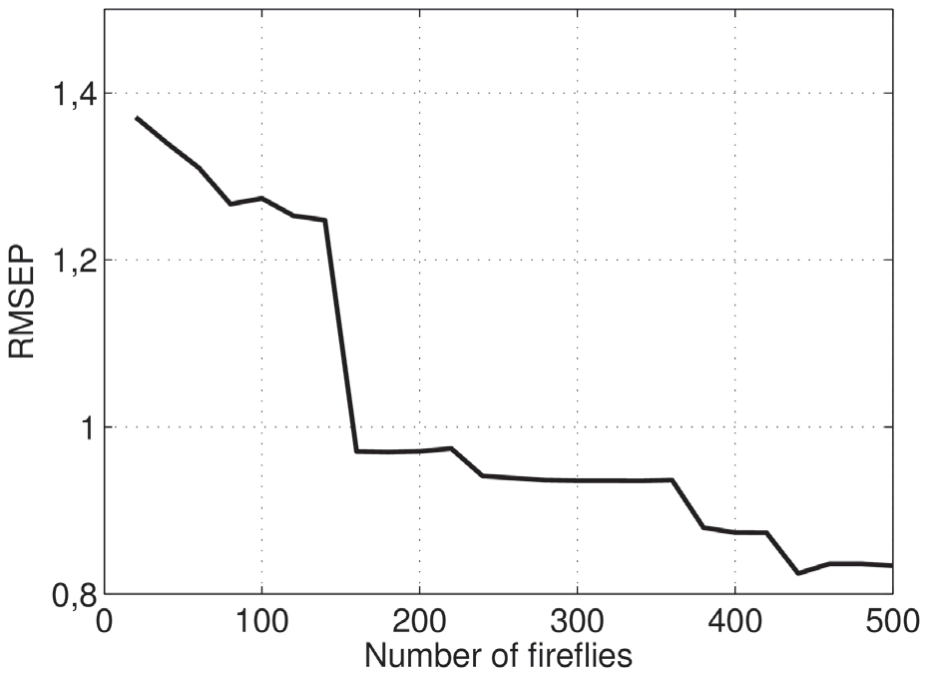
Behavior of RMSEP versus number of fireflies.

Fig. 2 presents how the number of fireflies affects the RMSEP. It is possible to note that we need a number between 400 and 500 fireflies to achieve the best results.

**Figure 3:**
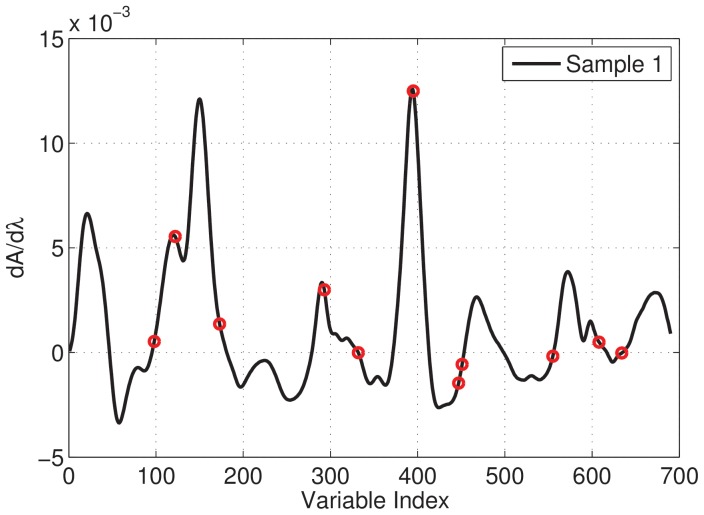
Visualization of selected variables.

The selected variables in the best firefly obtained can be visualized in Fig. 3. This result indicates that these regions are the most promising to use in the spectrophotometer. In practice, this result implies a smaller number of wavelengths measures in spectrophotometer for quantify the protein concentration property in real samples.

### Comparison with Traditional Algorithms

The comparison among the FA-MLR and traditional algorithms (SPA-MLR, GA-MLR, PLS and BVS) are presented in Table 6. As can be seen, the results of SPA-MLR, GA-MLR and PLS are relatively similar in terms of RMSEP, MAPE and PRESS. The results of BVS in terms of RMSEP and MAPE were relatively smaller in comparison to the results of SPA-MLR, GA-MLR and PLS. However, the FA-MLR has the lower RMSEP (0.09), MAPE (0.8%) and PRESS (4.08) using just 11 variables while BVS has 0.15, 1.07% and 6.96, of RMSEP, MAPE and PRESS, respectively, using 29 variables. The FA-MLR presents the lower AIC and BIC values, that is, the best parsimony. Indeed some scientists may prefer a smaller number of variables. In this case, the FA-MLR or PLS would be a more viable choice. However, despite to select a relatively small number of variables PLS uses all the original variables to build the new latent variables [55].

**Table 6:** Results of the FA-MLR, SPA-MLR, GA-MLR, PLS and BVS.

	Number of Variables	RMSEP	MAPE	AIC	BIC	PRESS
PLS	15	0.21	1.50%	1379	3630	10.09
SPA-MLR	13	0.20	1.43%	45.54	120.58	9.95
GA-MLR	146	0.21	1.50%	291.58	767.93	10.86
BVS	29	0.15	1.07%	49.70	131.26	6.96
FA-MLR	11	0.09	0.8%	31.45	96.34	4.08

**Figure 4:**
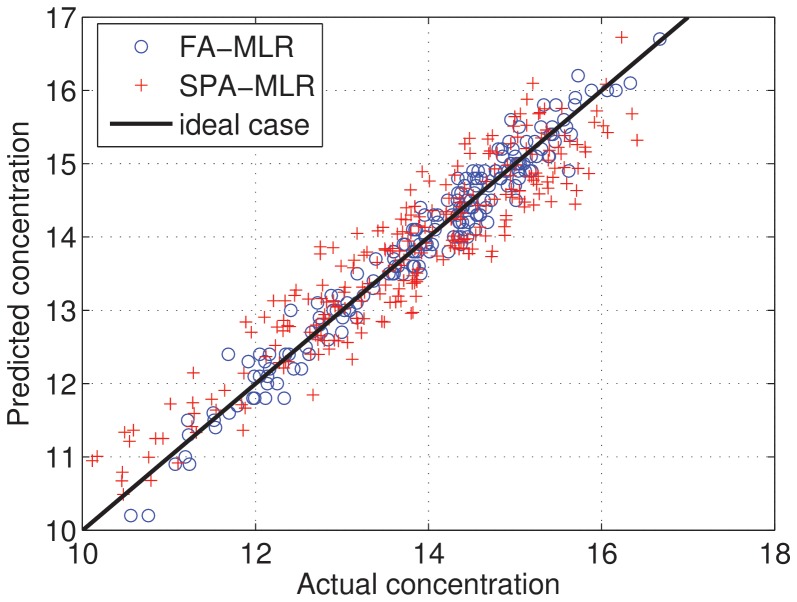
Comparison between actual and predicted concentration using the FA-MLR and the SPA-MLR.

Fig. 4 plots the real values in the compound versus predictions using the SPA-MLR (red plus) and FA-MLR (blue balls), that produced the best performance among the rival-tested algorithms. Zero differences between predictions and actual concentrations result in points over the straight line of the plot. The predicted concentrations are near the real concentrations for both methods. Nevertheless, the model using the variables selected by FA-MLR are in general closer to the straight line than SPA-MLR predictions. This result also indicates that the MLR model obtained using the variables selected by FA-MLR can produce less RMSEP and MAPE in average.

**Figure 5:**
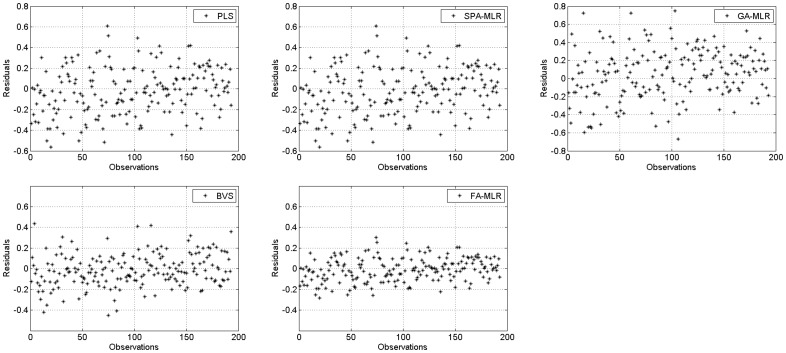
PRESS values for all algorithms: (a) PLS; (b) SPA-MLR; (c) GA-MLR; (d) BVS; and (e) FA-MLR.

Fig. 5 shows the PRESS values for all algorithms. To calculate the PRESS we used only the difference between the real (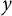) and predicted (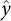) values of the property of interest. Using only the difference between 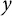 and 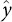 one can obtain a more exact residual value. It is possible to note that the PRESS values of the FA-MLR are more together and nearby from zero. This indicates that, in fact, the proposed FA-MLR is more robust to outliers observations and can provide a model with a better predictive ability.

In order to assess the sensitivity of the regression models to measurement noise, the matrix 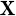 in the prediction set was contaminated with artificial zero-mean white gaussian noise. In each column, the standard deviation of the additional noise was set to 0.1 times the standard deviation of the actual value. As can be seen in Table 7, the results of FA-MLR and SPA-MLR remained largely unaltered after introduction of the artificial noise. On the other hand, the performance of GA-MLR, PLS and BVS was noticeably degraded, as compared to Table 6. Such a result can be ascribed to the fact that FA-MLR and SPA-MLR is aimed at minimizing multi-collinearity problems, which are known to increase the propagation of noise.

**Table 7:** Accuracy of regression models with artificial noise addition.

	RMSEP	MAPE
PLS	0.29	2.08%
SPA-MLR	0.23	1.64%
GA-MLR	0.35	2.51%
BVS	0.21	1.50%
FA-MLR	0.11	0.74%

### Speedup for FA-MLR using GPU

**Figure 6:**
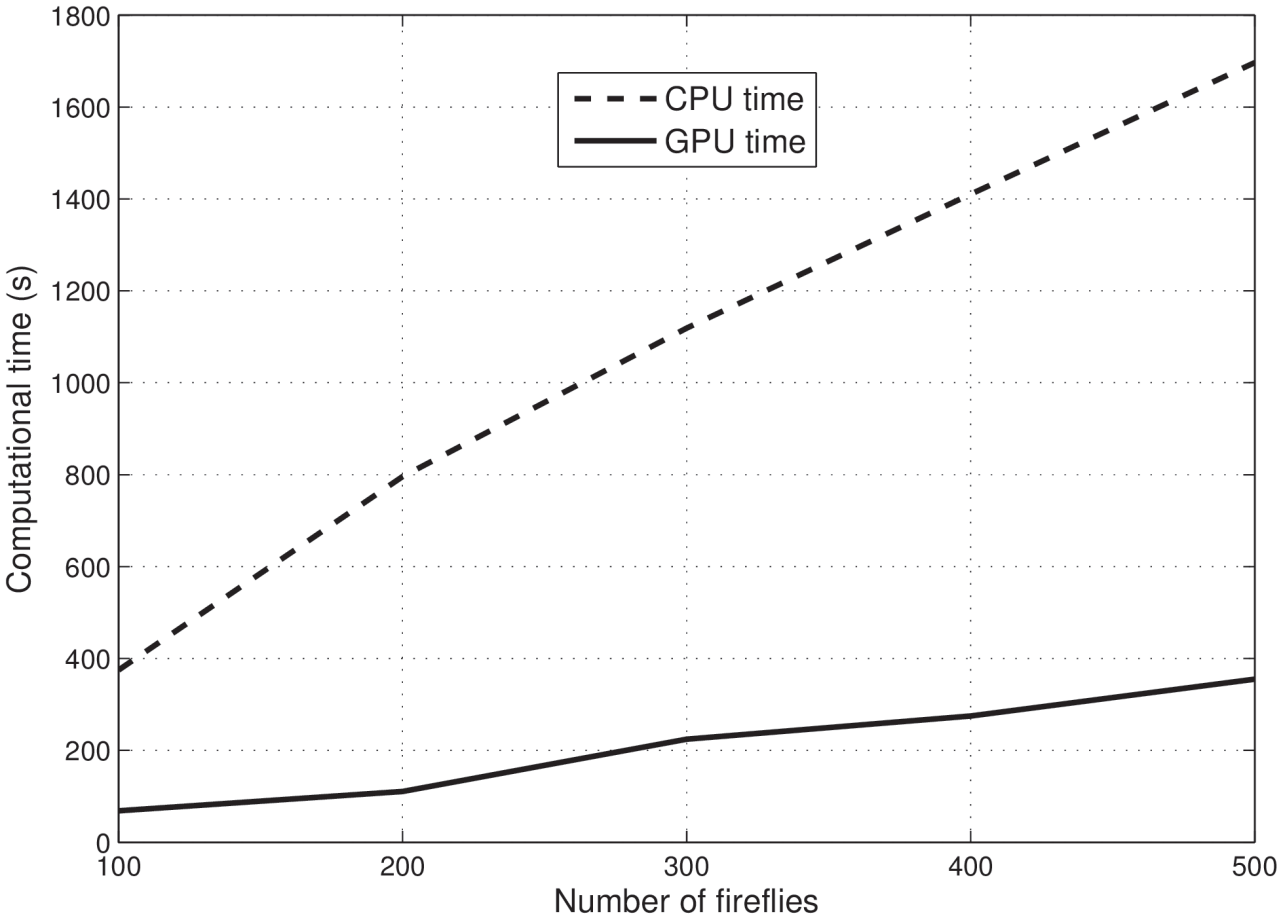
Comparison of computational performance between the FA-MLR using CPU and GPU.

Fig. 6 shows the computational performance of the FA-MLR execution using CPU and also GPU. Table 8 presents the comparison of computational time for FA-MLR according to the number of fireflies. The results showed that the FA-MLR using GPU is around five times faster than the FA-MLR using CPU.

**Table 8:** Computational time (seconds) for each implementation of the FA-MLR.

	Number of Fireflies		
	100	300	500
FA-MLR using GPU	69.34	223.56	355.34
FA-MLR using CPU	374.51	1119.63	1697.78

Table 9 presents a comparison of computational time between SPA-MLR, GA-MLR, PLS, BVS and FA-MLR according to the number of selected variables. It is possible to note that FA-MLR using GPU provides the lowest time.

**Table 9:** Computational time (seconds) for SPA-MLR, GA-MLR, PLS, BVS and FA-MLR.

	Time
PLS	2834.15
SPA-MLR	533.66
GA-MLR	372.22
BVS	315.68
FA-MLR using GPU	185.34
FA-MLR using CPU	931.45

The computational times in Table 9 represent the spent time for selecting up to 150 variables for SPA-MLR, GA-MLR, PLS and BVS, and using 250 fireflies for the FA-MLR. For PLS we have used the cross-validation technique to determine the number of latent variables, which explains the higher computational cost. Moreover, as can be seen the time spent for the proposed FA-MLR using GPU is the lowest in comparison with the others.

## Conclusions

Several studies have used the Firefly Algorithm (FA) to solve various types of problems. Based on the success of such works, one found out that using a modified FA is also possible to select variables in multivariate calibration problems. Thus, this paper presented a GPU-based FA (FA-MLR) for selection of variables in protein determination problem in whole grain wheat samples. In addition a variable selection problem in simulated study was presented.

Using a multiobjective formulation of FA, our implementation was able to perform the reduction of all measures in all tests. In terms of RMSEP, PRESS, AIC and BIC the FA-MLR showed the lower values while compared with SPA-MLR, GA-MLR, PLS and BVS. Furthermore, the FA-MLR using GPU showed a significant speedup in relation to its CPU implementation. The speedup gain was 5.25x. Therefore, the FA-MLR would be a more suitable implementation for the problem of variable selection in multivariate calibration problems.

In future works, larger multivariate calibration problems may be solved. In addition, alternatives to CUDA-MATLAB integration such as OpenCL could be investigated for comparative studies.

## Supporting Information

S1 Source Code (DOCX)

## References

[1] Brown SD, Blank TB, Sum ST, Weyer LG (1994) Chemometrics. Analytical chemistry 66:315–359. 10.1021/ac00084a014 8092471

[2] Ferreira MMC, Antunes AM, Melgo MS, Volpe PLO (1999) Quimiometria I: calibracao multivariada, um tutorial. Quimica Nova 22:724–731.

[3] George EI (2000) The variable selection problem. Journal of the American Statistical Association 95:1304–1308.

[4] Coifman RR, Wickerhauser MV (1992) Entropy-based algorithms for best basis selection. Information Theory, IEEE Transactions on 38:713–718.

[5] Paula LCM, Soares AS, Lima TW, Martins WS, Filho ARG, et al**.** (2013) Partial parallelization of the successive projections algorithm using compute unified device architecture. In: Internation Conference on Parallel and Distributed Processing Techniques and Applications. pp.737–741.

[6] Chau FT, Liang YZ, Gao J, Shao XG (2004) Chemometrics: from basics to wavelet transform, volume 234. Wiley. com.

[7] Galvão Filho AR, Galvão RK, Araújo MCU (2011) Effect of the subsampling ratio in the application of subagging for multivariate calibration with the successive projections algorithm. Journal of the Brazilian Chemical Society 22:2225–2233.

[8] Lucena DV, Lima TW, Soares AS, Delbem ACB, Galvao AR, et al**.** (2013) Multi-objective evolutionary algorithm for variable selection in calibration problems: A case study for protein concentration prediction. In: Proceedings of 2013 IEEE Congress on Evolutionary Computation. pp.1053–1059.

[9] Yang XS (2008) Nature-inspired metaheuristic algorithms. Luniver Press.

[10] Yang XS (2009) Firefly algorithms for multimodal optimization. In: Stochastic algorithms: foundations and applications, Springer. pp.169–178.

[11] Lukasik S, Zak S (2009) Firefly algorithm for continuous constrained optimization tasks. In: Computational Collective Intelligence. Semantic Web, Social Networks and Multiagent Systems, Springer. pp.97–106.

[12] Yang XS (2010) Firefly algorithm, stochastic test functions and design optimisation. International Journal of Bio-Inspired Computation 2:78–84.

[13] Senthilnath J, Omkar S, Mani V (2011) Clustering using firefly algorithm: Performance study. Swarm and Evolutionary Computation 1:164–171.

[14] Gandomi AH, Yang XS, Alavi AH (2011) Mixed variable structural optimization using firefly algorithm. Computers & Structures 89:2325–2336.

[15] Jati GK, Suyanto (2011) Evolutionary discrete firefly algorithm for travelling salesman problem. In: Adaptive and Intelligent Systems, Springer. pp.393–403.

[16] Banati H, Bajaj M (2011) Fire fly based feature selection approach. International Journal of Computer Science Issues 8:473–480.

[17] Horng MH (2012) Vector quantization using the firefly algorithm for image compression. Expert Systems with Applications 39:1078–1091.

[18] Abdullah A, Deris S, Anwar S, Arjunan SN (2013) An evolutionary firefly algorithm for the estimation of nonlinear biological model parameters. PloS one 8:e56310. 23469172 10.1371/journal.pone.0056310 PMC3587642

[19] Husselmann AV, Hawick K (2012) Parallel parametric optimisation with firefly algorithms on graphical processing units. In: Proc. Int. Conf. on Genetic and Evolutionary Methods (GEM12). Number CSTN-141, Las Vegas, USA, CSREA (16–19 July 2012). pp.77–83.

[20] Husselmann A, Hawick K (2014) Geometric firefly algorithms on graphical processing units. In: Cuckoo Search and Firefly Algorithm, volume 516 . pp.245–269.

[21] Soares AS, Delbem ACB, Lima TW, Coelho CJ, Soares FAAMN (2013) Mutation-based compact genetic algorithm for spectroscopy variable selection in the determination of protein in wheat grain samples. Eletronic Letters 49:80–92.

[22] Soares AS, de Lima TW, Soares FAAMN, Coelho CJ, Federson FM, et al (2014) Mutation-based compact genetic algorithm for spectroscopy variable selection in determining protein concentration in wheat grain. Electronics Letters 50:932–934 (2)..

[23] Haaland DM, Thomas EV (1988) Partial least-squares methods for spectral analyses. 1. relation to other quantitative calibration methods and the extraction of qualitative information. Analytical Chemistry 60:1193–1202.

[24] Carbonetto P, Stephens M (2012) Scalable variational inference for bayesian variable selection in regression, and its accuracy in genetic association studies. Bayesian Analysis 7:73–108.

[25] Martens H (1991) Multivariate calibration. John Wiley & Sons.

[26] Westad F, Martens H (2000) Variable selection in near infrared spectroscopy based on significance testing in partial least squares regression. Journal of Near Infrared Spectrosc 8:117–124.

[27] Naes T, Mevik BH (2001) Understanding the collinearity problem in regression and discriminant analysis. Journal of Chemometrics 15:413–426.

[28] Cortina JM (1994) Interaction, nonlinearity, and multicollinearity: Implications for multiple regression. Journal of Management 19:915–922.

[29] Beebe KR, Pell RJ, Seasholtz MB (1998) Chemometrics: a practical guide. Wiley-Interscience Series on Laboratory Automation, 341 pp.

[30] Lawson CL, Hanson RJ (1974) Solving least squares problems, volume 161. SIAM

[31] Hibon M, Makridakis S (1995) Evaluating accuracy (or error) measures. INSEAD

[32] Allen DM (1974) The relationship between variable selection and data agumentation and a method for prediction. Technometrics 16:125–127.

[33] Tarpey T (2000) A note on the prediction sum of squares statistic for restricted least squares. The American Statistician 54:116–118.

[34] Bartoli A (2009) On computing the prediction sum of squares statistic in linear least squares problems with multiple parameter or measurement sets. International journal of computer vision 85:133–142.

[35] Box GEP, Jenkins GM, Reinsel GC (2013) Time series analysis: forecasting and control. Wiley.com.

[36] Akaike H (1974) A new look at the statistical model identification. Automatic Control, IEEE Transactions on 19:716–723.

[37] Schwarz G (1978) Estimating the dimension of a model. The annals of statistics 6:461–464.

[38] Yang XS, He X (2013) Firefly algorithm: recent advances and applications. International Journal of Swarm Intelligence 1:36–50.

[39] de Lucena DV, de Lima TW, da Silva Soares A, Coelho CJ (2012) Multi-objective evolutionary algorithm nsga-ii for variables selection in multivariate calibration problems. International Journal of Natural Computing Research 3:43–58.

[40] Anderson da Silva Soares AdS, de Lima TW, LuPcena DVd, Salvini RL, Laureano GT, et al (2013) Spectroscopic multicomponent analysis using multiobjective optimization for variable selection. Computer Technology and Application 4:465–474.

[41] Yang XS (2013) Multiobjective firefly algorithm for continuous optimization. Engineering with Computers 29:175–184.

[42] CUDA N (2011) NVIDIA CUDA C Programming Guide. 2701 San Tomas Expressway Santa Clara, CA 95050: NVIDIA Corporation, 4.0 edition.

[43] Luebke D, Humphreys G (2007) How gpus work. Computer 40:96–100.

[44] Tsuchiyama R, Nakamura T, Iizuka T, Asahara A, Son J, et al**.** (2010) The OpenCL Programming Book. Fixstars.

[45] Little J, Moler C**.** Matlab gpu computing support for nvidia cuda-enabled gpus. Available: http://www.mathworks.com/discovery/matlab-gpu.html. Accessed 2013 Nov 3.

[46] *MathWorks* **.** Matlab gpu computing support for nvidia cuda-enabled gpus. Available: http://www.mathworks.com/discovery/matlab-gpu.html. Accessed 2013 Nov 3.

[47] Reese J, Zaranek S**.** Gpu programming in matlab. Available: http://www.mathworks.com/company/newsletters/articles/. Accessed 2013 Nov 3.

[48] Liu X, Cheng L, Zhou Q (2013) Research and comparison of cuda gpu programming in matlab and mathematica. In: Proceedings of 2013 Chinese Intelligent Automation Conference. Springer, pp.251–257.

[49] Araújo MCU, Saldanha TCB, Galvão RKH, Yoneyama T, Chame HC, et al (2001) The successive projections algorithm for variable selection in spectroscopic multicomponent analysis. Chemometrics and Intelligent Laboratory Systems 57:65–73.

[50] Kennard RW, Stone LA (1969) Computer aided design of experiments. Technometrics 11:137–148.

[51] Soares AS, Galvao RKH, Araujo MCU, Soares SFC, Pinto LA (2010) Multi-core computation in chemometrics: case studies of voltammetric and nir spectrometric analyses. Journal of the Brazilian Chemical Society 21:1626–1634.

[52] Savitzky A, Golay MJ (1964) Smoothing and differentiation of data by simplified least squares procedures. Analytical chemistry 36:1627–1639. 10.1021/ac60319a045 22324618

[53] Bradstreet RB (1965) The Kjeldahl method for organic nitrogen.

[54] Galvao RKH, Araujo MCU, Fragoso WD, Silva EC, Jose GE, et al (2008) A variable elimination method to improve the parsimony of {MLR} models using the successive projections algorithm. Chemometrics and Intelligent Laboratory Systems 92:83–91.

[55] Tobias RD (1995) An introduction to partial least squares regression. In: Proceedings of Ann. SAS Users Group Int. Conf., 20th, Orlando, FL. pp.2–5.

