# Supplementary material for: A GPU-Based Implementation of the Firefly Algorithm for Variable Selection in Multivariate Calibration Problems
Source: PLoS One. 2014 Dec 10;9(12):e114145. doi: 10.1371/journal.pone.0114145 (PMC4262411; doi:10.1371/journal.pone.0114145)
Supplement: S1 Source Code — (DOCX) [file pone.0114145.s001.docx]

%Source code to generate the simulated data set

randn('seed',0);

NSamples = 100;

Nvars = 200;

Number_of_variables_correlated_X_Y = 5;

X = randn(NSamples,Nvars);

vars = randperm(size(X,1));

vars = vars([1:Number_of_variables_correlated_X_Y]);

Y = X(:,vars)*randn(1,size(vars,2))';

indice_amostras = randperm(size(X,1));

size_train = round(size(X,1)*0.6);

size_Test = round(size(X,1) - size_train);

samples_Train = indice_amostras([1:size_train]);

samples_Test = indice_amostras([size_train+1:size_train+round(size_Test/2)]);

samples_Pred = indice_amostras([size_train+round(size_Test/2)+1:size(X,1)]);

Xtrain = X(samples_Train,:);

Ytrain = Y(samples_Train,:);

Xtest = X(samples_Test,:);

Ytest = Y(samples_Test,:);

Xpred = X(samples_Pred,:);

Ypred = Y(samples_Pred,:);
